# The ligand binding mechanism to purine nucleoside phosphorylase elucidated via molecular dynamics and machine learning

**DOI:** 10.1038/ncomms7155

**Published:** 2015-01-27

**Authors:** Sergio Decherchi, Anna Berteotti, Giovanni Bottegoni, Walter Rocchia, Andrea Cavalli

**Affiliations:** 1 CONCEPT Lab, Istituto Italiano di Tecnologia, via Morego 30, 16163 Genova, Italy; 2 BiKi Technologies s.r.l., via XX Settembre 33, 16121 Genova, Italy; 3 CompuNet, Istituto Italiano di Tecnologia, via Morego 30, 16163 Genova, Italy; 4 Department of Pharmacy and Biotechnology, University of Bologna, via Belmeloro 6, 40126 Bologna, Italy

## Abstract

The study of biomolecular interactions between a drug and its biological target is of paramount importance for the design of novel bioactive compounds. In this paper, we report on the use of molecular dynamics (MD) simulations and machine learning to study the binding mechanism of a transition state analogue (DADMe–immucillin-H) to the purine nucleoside phosphorylase (PNP) enzyme. Microsecond-long MD simulations allow us to observe several binding events, following different dynamical routes and reaching diverse binding configurations. These simulations are used to estimate kinetic and thermodynamic quantities, such as *k*_on_ and binding free energy, obtaining a good agreement with available experimental data. In addition, we advance a hypothesis for the slow-onset inhibition mechanism of DADMe–immucillin-H against PNP. Combining extensive MD simulations with machine learning algorithms could therefore be a fruitful approach for capturing key aspects of drug–target recognition and binding.

## Abstract (web summary)

Understanding the dynamics of enzyme-substrate complexation provides an insight into potential drugs, but intermediate states are difficult to observe experimentally. Here, the authors use simulations and machine learning to analyse the binding of transition state inhibitors to purine nucleoside phosphorylase.

## Graphical abstract

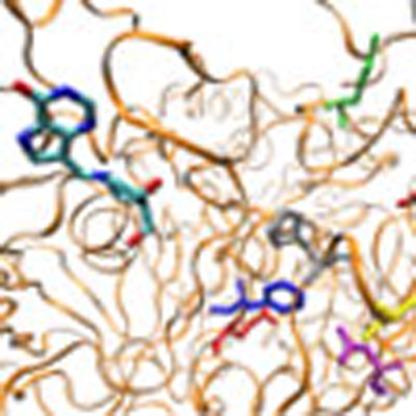

Protein–ligand binding is at the basis of the therapeutic effect of drugs, and understanding this process is of paramount importance in drug discovery^1^. Disclosing atomistic details of protein–ligand binding can help the rational design of bioactive compounds, while predicting the free energy and kinetics associated with this process can help prioritize drug candidates^2^. In this context, the role of biomolecular modelling and, more specifically, of molecular dynamics (MD) has been widely accepted and also recently recognized^3^. Nowadays, it is possible to extensively simulate systems of several hundred thousand atoms, characterizing many mechanistic aspects of the protein–ligand binding process. These investigations benefit from the unprecedented computational power of new generation computer architectures, and from software tools that can exploit these innovative hardware infrastructures. In a few recent cases, computational approaches employing massively parallel architectures provided a full dynamical description of protein–ligand binding, affording estimations of free energy and kinetics associated with the process, with various degrees of accuracy^4^,^5^,^6^,^7^,^8^.

Transition state analogues (TSAs) are chemical structures that resemble enzymatic transition states in terms of geometric and electrostatic features. TSA ligands are among the most powerful enzymatic inhibitors ever discovered, and several TSAs are currently in clinical trials^9^. Notable examples include the TSA inhibitors of human purine nucleoside phosphorylase (PNP), a homotrimeric enzyme that catalyzes the reversible phosphorolysis of 6-oxopurine nucleosides and deoxynucleosides to the corresponding purine base and α-d-(deoxy)ribose 1-phosphate^10^. PNP deficiency elevates d-guanosine concentrations in the blood, resulting in apoptosis of dividing T cells due to the accumulation of dGTP, an inhibitor of ribonucleotide reductase^11^. Inhibitors of PNP can be used to treat T-cell cancers and autoimmune diseases including gout, rheumatoid arthritis, psoriasis, tissue transplant rejection and multiple sclerosis^11^. TSA inhibitors of PNP include immucillin-H (now in phase II clinical trials under the name of forodesine) and DADMe–immucillin-H (passed phase IIb clinical trial under the name of ulodesine), which were discovered by Schramm and co-workers^11^. The same group carried out very thorough experimental analyses to characterize the structural determinants of the interaction as well as the kinetics of PNP inhibition by TSA ligands. In addition, several crystal structures have been determined for PNP, both in the apo and holo forms (examples of PDB entries are: 3K80, 1RSZ, 1M73 and 3BGS)^11^, providing a large amount of data related to TSA ligands in complex with this enzyme. However, the mechanism of a TSA inhibitor binding to PNP has not so far been elucidated at the atomistic level. From the computational standpoint, PNP is a challenging and relatively big system (about 100,000 atoms, including solvent). Importantly, several kinetic rate constants related to potent PNP-TSA inhibitors are currently available^11^. As such, we considered PNP–TSA complexes to be optimal test beds for newly developed computational tools and theories.

In this work, we integrate microsecond-long MD simulations with machine learning algorithms to identify the main structural and dynamical features of a TSA inhibitor binding to PNP. We implement a k-medoids clustering method^12^ to extract a set of human-interpretable and meaningful mesostates from long MD trajectories. The protocol is fully automated, as the number of clusters is automatically detected and requires no user intervention. In detail, we investigate the binding mechanism of DADMe–immucillin-H (hereafter referred to as DADME) to the PNP enzyme^11^, also providing the free energy profiles along the binding routes^13^. DADME is a tight binding TSA inhibitor of PNP, showing an inhibition constant (*K*_i_) of 9 pM and a residence time (the inverse of the *k*_off_) of 20 min^11^. We show that our protocol is able to identify several routes for DADME binding to PNP and to characterize all the routes from the mechanistic and energetic standpoints. Interestingly, DADME binding to PNP shows an unexpected binding path and quite unique features, including a slow-onset inhibition^14^. The binding process simulated here is in full agreement with the available experimental findings^15^. The bound state, as obtained via microsecond-long MD simulations, is remarkably similar to the crystallographic structure of PNP in complex with DADME (including structures having RMSD <0.6 Å with respect to the crystal). In addition, we obtain an estimate of the binding *k*_on_, which is in fairly good agreement with experimental data. Scaled MD^16^ is used to investigate the unbinding mechanism and elucidate details related to the DADME–PNP dissociation process. Based on this dynamical picture, we advance a novel hypothesis of the slow-onset tight binding inhibition mechanism of DADME towards PNP.

## Results

### Structure analysis and binding simulation

**Figure 1:**
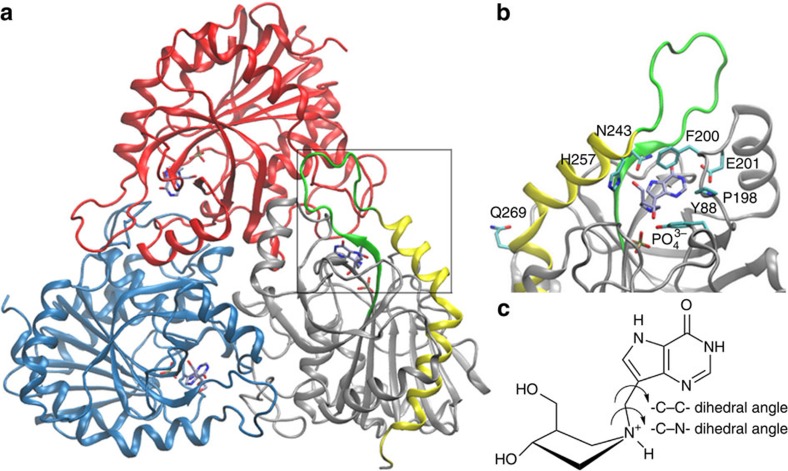
Structures of the human PNP trimer and DADMe–immucillin-H. (**a**) PNP trimeric structure (PDB entry 3K8O): monomers corresponding to chains E, Q and Y are represented as ribbons in silver, blue and red, respectively. The catalytic sites are rather close to the interface between different monomers and can be recognized by the presence of the ligand and phosphate molecules. (**b**) Larger view of the PNP active site in the bound state with DADMe–immucillin-H (PDB entry 1RSZ). The main residues interacting with the ligand as well as the phosphate molecule and the ligand itself are represented in licorice mode. In green is shown the so-called ‘gate’ loop, formed by residues 240–256, and in yellow the α-helix, which makes intermittent contacts with the bound ligand and which is kinked at Gln269. (**c**) Two-dimensional structure of DADMe–immucillin-H. The torsional angles that are discussed in the text are explicitly reported.

In Fig. 1, we report the PNP trimer (Fig. 1a) along with a Two-dimensional sketch of DADME chemical structure (Fig. 1c). In our structural analysis, we will comment in particular on some key residues that we refer to as the binding site residues, namely His257, Asn243, Phe200, Pro198, Glu201 and Tyr88 (PDB entry 1RSZ, see Fig. 1a,b). A relevant secondary structure element is the α-helix made by residues 257–284. This α-helix is kinked at Gln269. A loop (residues 240–256) is located right before this α-helix. We will refer to it as the gate. The ligand comprises a purine and a dihydroxypyrrolidine ring connected via two bonds (see Fig. 1c). By flipped pose we mean a DADME conformation where the -C–N- dihedral angle between the dihydroxypyrrolidine and the purine ring differs from that of the crystallographic structure by about 120°.

**Figure 2:**
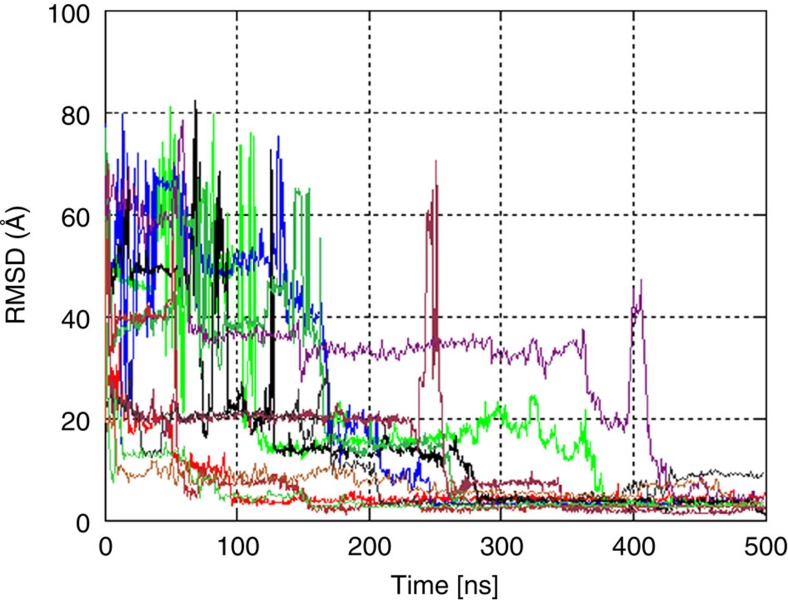
RMSD of DADMe–immucillin-H with changing simulation time. Non-hydrogen atom displacement was monitored over the simulation time relative to the crystal structure. Colour encodes the simulation runs. All the simulations reported in the plot led to binding.

In our simulation set-up, a PNP trimer, the biological functional unit, and 9 ligands freely evolve in a cubic box full of explicit water molecules, summing up to about 100,000 atoms. Out of 14 runs, we identified 11 events that can be ascribed to binding. To monitor the binding process, we used the root mean square deviation (RMSD) of the heavy atoms of the ligand after superimposing the backbone binding site residues onto the reference structure, represented by the PNP monomer in complex with DADME (PDB entry 1RSZ) (Fig. 2).

**Figure 3:**
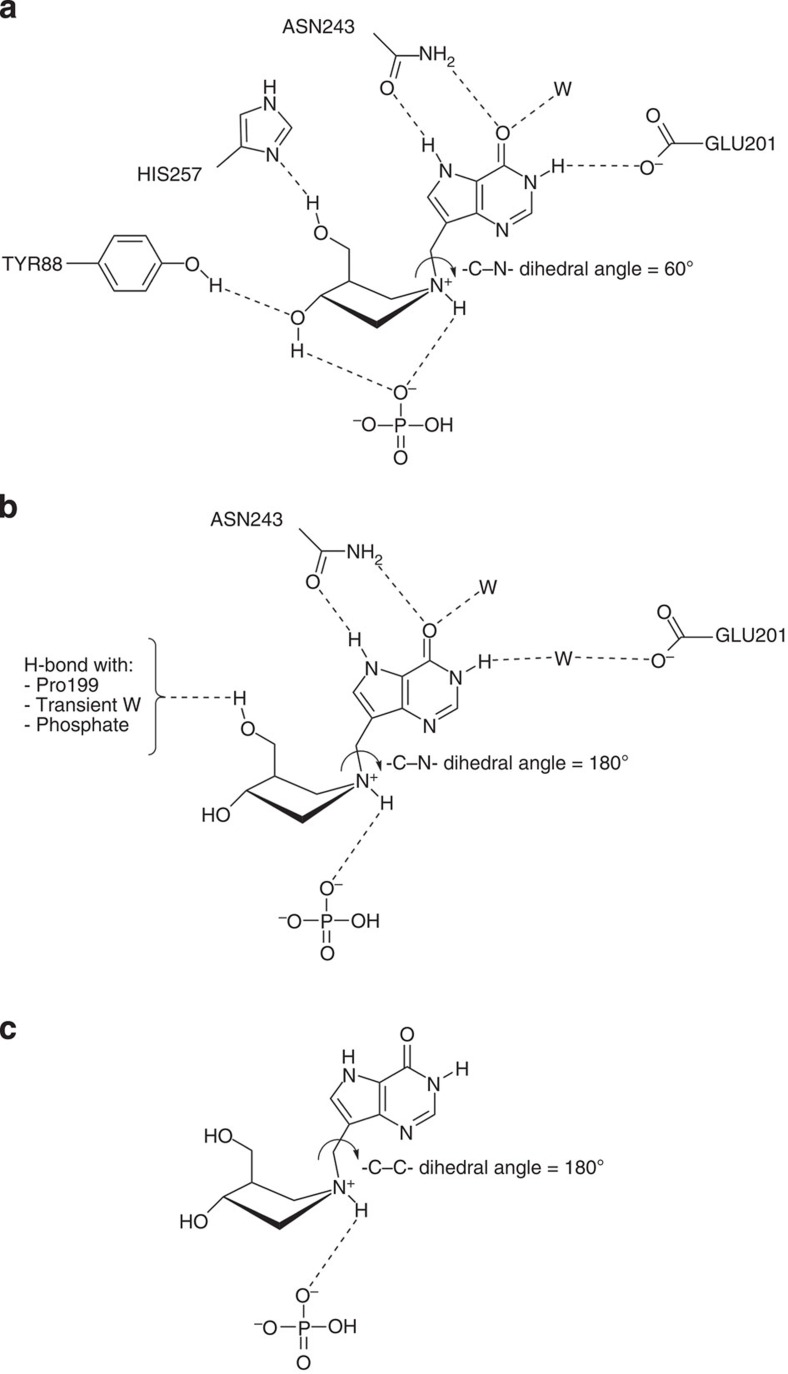
DADME binding ensembles. (**a**) Ensemble A representing the crystallographic conformation, which was correctly recovered by our simulations. (**b**) Ensemble B characterized by a rotation of 120 degrees of the -C–N- dihedral angle compared with the crystallographic structure. (**c**) Ensemble C characterized by a rotation of 180 degrees, compared with the crystallographic structure, of -C–C- dihedral angle. W, water molecule.

In three runs, the RMSD reached values <1.25 Å (hereafter referred to as ensemble A or state A, see Fig. 3a). In one of these, the binding was direct, namely the ligand reached the final tight binding conformation without spending a significant amount of time in intermediate mesostates. In the other two, the final configuration was achieved after stopping over into a few intermediate mesostates. In another four runs, the final RMSD ranged between 2 and 3 Å (hereafter referred to as ensemble B or state B, see Fig. 3b). In four runs, the RMSD of the structures in the final metastable state was between 3 and 4 Å (hereafter referred to as ensemble C or state C, see Fig. 3c).

To further validate our approach, the same MD protocol was carried out using PNP and acyclovir, a much weaker inhibitor relative to DADME (*K*_i_=90 μM). Notably, out of five simulations, only one reached a stable binding configuration, with an RMSD versus the X-ray structure (PDB entry 1PWY; ref. 17) of 2.8 Å. Conversely, all the other runs led to final configurations showing an RMSD >4 Å from the crystal structure (see Methods section and Supplementary Discussion for further details on these simulations).

The set of conformations referred to as ensemble A consists of structures that strictly resemble the crystallographic one, achieving an RMSD as low as 0.59 Å. In detail, the 3′-OH of DADME H-bonds the phosphate group, and Asn243 stabilizes the ligand by a bi-dentate interaction with the purine ring (Fig. 3a). Kicska *et al*.^18^ have reported that breaking the bi-dentate interaction could lead to a change in the binding free energy of about 10 kcal mol^−1^. Based on the present calculations and previous experimental observations, we point to this interaction as a key element of the entire binding process. In addition, the π–π stacking between the purine ring of DADME and the phenyl ring of Phe200 is another persistent feature that we observed during binding. These interactions embed the purine ring of DADME into the PNP catalytic pocket, whereas the dihydroxypyrrolidine remains more solvent exposed. As it can be seen in Supplementary Fig. 2, we did not observe a strong persistency of the H-bond between the 5′-OH group of DADME and His257, in agreement with other data previously reported in the literature^10^.

The main characterizing feature of the ensemble B structures is a flip of 120° of the -C–N- dihedral angle between purine and dihydroxypyrrolidine rings, when compared to the crystallographic structure (Fig. 3b, see also Supplementary Fig. 3 for a free energy characterization). While the bi-dentate interaction with Asn243 is maintained, the 3′-OH of DADME often interacts via H-bond either with the inorganic phosphate (as in the crystal structure) or, mediated by a water molecule, with Pro198 (see Supplementary Fig. 4 for the ligand hydration vs time during the binding process). We observed that the flipped pose could inter-convert to the crystallographic conformation leading to the ensemble A (see Supplementary Movie 1). In addition, as with ensemble A, the propensity of the α-helix to kink and close the binding site was rather low. Similarly, in the simulations leading to the flipped pose, a water molecule often acted as a substitute of the phosphate by H-bonding one -OH of the ligand and stabilizing the dihydroxypyrrolidine.

In the set of configurations referred to as ensemble C the bi-dentate interaction with Asn243 is missing, and the purine ring is rotated by about 180° with respect to the crystal pose (-C–C- dihedral angle; see Fig. 3c). This configuration was quite stable during the simulations, mainly thanks to van der Waals interactions. From ensemble C, DADME could either evolve to B or escape from the enzyme towards the solvent.

### Identifying binding paths and intermediates

**Figure 4:**
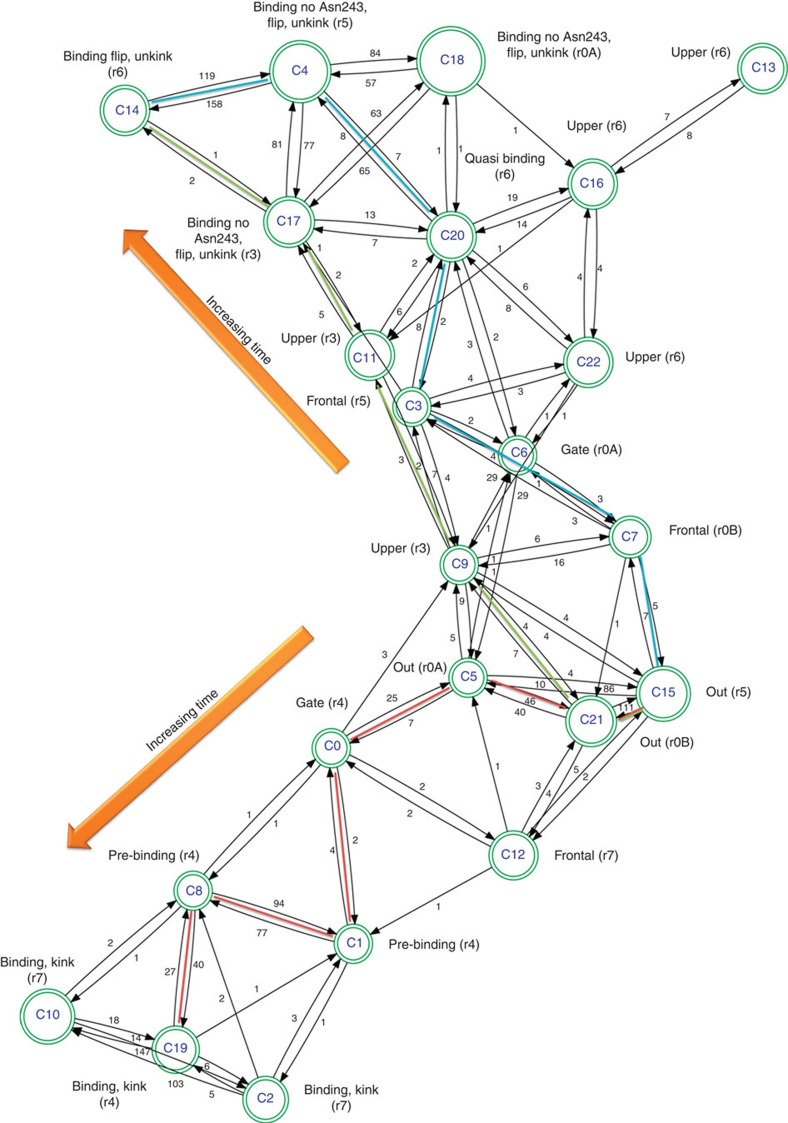
Clustering of all the trajectories via k-medoids algorithm. Circle sizes encode the cluster sizes. On each edge, the number of transitions between connected clusters is reported. Legends provide a synthetic description of the state and the numbers between braces indicate the replica to which the medoid of the corresponding cluster belongs. The colours encode the shortest paths identified as binding routes. Starting from the centre (out state) time increases along centrifugal directions.

To atomistically characterize the binding paths and find relevant intermediates, we developed an *ad hoc* protocol. In Fig. 4, we show the binding scheme as obtained by clustering all the trajectories that were generated on 13 μs of MD simulations (see the Methods section for further details). Each cluster was labelled according to the conformation of its medoid. To obtain the binding paths, we ran the Dijsktra shortest path algorithm on the clustering graph using as edge weights the negative logarithm of the number of transitions (a quantity reminiscent of the free energy). The starting node was labelled ‘out’ (cluster 15), while the ending nodes were ensembles A (cluster 19), B (cluster 14) and C (cluster 4). Then, we iteratively computed the shortest path and removed the visited nodes until the destination node became unreachable. Several algorithms can be used to find sub-optimal shortest path in a graph (see for instance the WISP algorithm^19^). The procedure here employed shows, however, some useful features, which are here summarized. The number of paths is automatically detected and the path identification is extremely fast. In addition, it selects paths that do not have any node (that is, cluster) in common and thus can be considered as ‘independent’.

**Figure 5:**
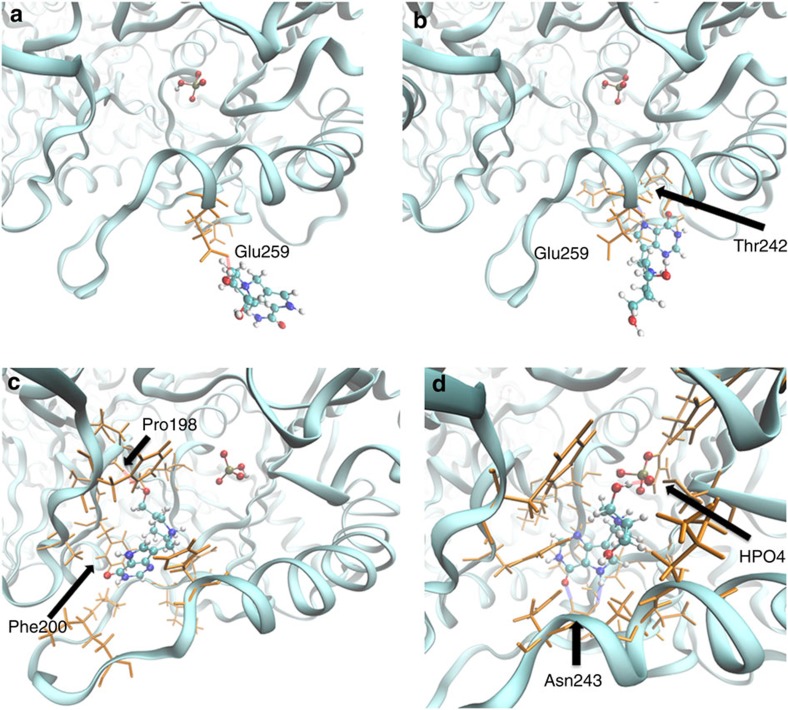
Structural representation of intermediate binding configurations along the gating mechanism. (**a**) DADME (in CPK representation) on the PNP surface. No specific or transient interactions with Glu259 were identified at this stage of binding. (**b**) DADME interacting with PNP before gate opening and entrance into the enzyme. At this stage, an H-bond with Thr242 and a transient interaction with Glu259 could be identified. (**c**) DADME entering the binding site of PNP right after the gate opening. Here the ligand is quite well stabilized by specific interactions with Pro190 (H-bond) and with Phe200 (parallel π–π stacking). (**d**) DADME into the PNP binding pocket assuming the conformation of the bound state, that is, that observed in ensemble A (see Fig. 3).

Three different binding routes were obtained and named upper, frontal and gating (see Supplementary Movies 2–4 for representative movies of each binding route and Fig. 5 for representative configurations of the entrance via the gating mechanism. See also Supplementary Figs 6,7, and 8 for the clustering of every single observed path). Notably, there was not an exclusive relationship between entrance pathways and final ensembles, that is, each binding route could lead to ensemble A, B, or C. Upper and frontal routes were intuitive and quite similar: in both cases, the α-helix facing the binding site partially lost its kink and allowed the ligand to enter the binding site either from above the phosphate or from a ‘frontal’ entrance, located at the interface between two monomers. The third binding path (gating) was somewhat unexpected: the ligand passed through a gap between the α-helix and the loop facing the binding site. This passage did not always require the α-helix to lose its kink. The gating route led to the final binding configuration state, where an RMSD of 0.59 Å versus the crystallographic structure was observed.

In the case of the upper entrance, the ligand entered the binding site only when the α-helix left enough space and the site was exposed. The first observed interactions were between the ligand dihydroxypyrrolidine and the phosphate. This detail was captured by clusters 9 and 11 (see Fig. 4 and Supplementary Fig. 5). Then, DADME established hydrophobic interactions with Phe200. Minor variations of the final binding configuration differed by slight conformational changes of Phe200 or Tyr88 side chains, which stabilized DADME through π–π interactions. His257 was observed playing a role in the early stage of this path, by stabilizing the purine ring (for further details on the role of His257 during binding, see the Supplementary Discussion).

The frontal entrance is probably the most obvious entry point, located at the edge of two adjacent monomers. Here too, we observed His257 making transient interactions with DADME before reaching the binding site. In one of the trajectories, the ligand bound Phe159 instead of His257. An invariant feature of the frontal routes was an H-bond between Asp157 and the -OH or -NH of the dihydroxypyrrolidine ring. During the entrance, the -OH of DADME established an H-bond with Asp157, whereas the purine interacted via cation-π with Arg154 and the -NH of dihydroxypyrrolidine H-bonded to Phe155 oxygen. We found that the -OH of the dihydroxypyrrolidine H-bonded to Asp109, and Arg24 established cation-π interaction with the purine ring of the ligand.

In a further path here identified, we observed DADME entering via a gating mechanism. Notably, this mechanism was previously hypothesized by comparing apo and holo crystal structures of PNP^20^, pointing to residues 241–260 as key amino acids that should move to allow ligand entering into the protein. In detail, DADME formed transient H-bonds with Glu259, Glu253 and Ans243 before a major rearrangement opened the gate as shown in the Supplementary Movie 4. Figure 5 captures key steps of the gating mechanism: Fig. 5a represents the very first approach of the ligand towards the gate, then, in Fig. 5b, the ligand is trapped by amino acids 240–256, while in Fig. 5c,d, the ligand respectively overcomes the gate and reaches the final binding pose, corresponding to the crystallographic conformation (that is, ensemble A). Notably, the gating mechanism led to a configuration with an RMSD of 0.59 Å relative to the PNP–DADME crystallographic structure (ensemble A; cluster 19 in Fig. 4). In a different trajectory, we observed DADME entering into PNP only when the Val260 side chain rotated by 100° compared with the apo crystal structure. During the pre-binding phase, the side chain of Val260 hampered the formation of the pivotal interaction between DADME and Asn243. A mandatory conformational rearrangement of Val260 allowed DADME to reach the final binding configuration, corresponding to ensemble A. On binding, Val260 recovered the crystallographic conformation.

### Free energy estimation along the binding paths

We then characterized the binding paths in terms of free energy. To this aim, we performed umbrella sampling simulations along the binding routes using the path collective variable^21^ (S-variable) as reaction coordinate. The free energy was then reconstructed using a non-linear regression algorithm, via an approach similar to that reported by Maragliano *et al*.^22^ (see Methods for further details).

**Figure 6:**
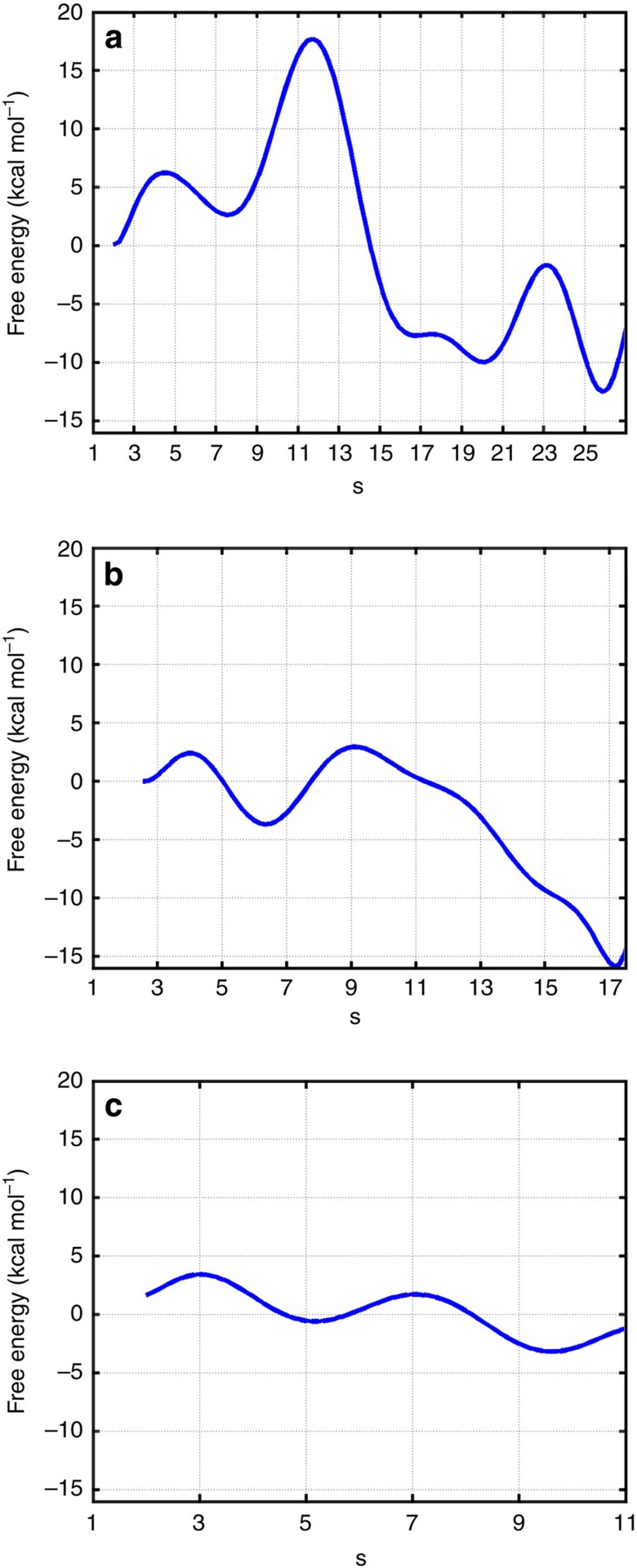
Free energy estimation for the three different binding paths towards different binding poses. (**a**) Free energy path associated to the gating mechanism leading to ensemble A. (**b**) Free energy path associated to the frontal mechanism leading to ensemble A. (**c**) Free energy path associated to the upper mechanism leading to ensemble C. When leading to the crystallographic pose (ensemble A) the overall free energy difference between bound and unbound is about 13–14 kcal mol^−1^, whereas ensemble C is a kind of loose pre-binding state. Gating and frontal are well characterized paths, while the upper path exhibits a less featured profile.

In Fig. 6, the free energy profiles along the three paths (gating, frontal and upper) are reported. The gating mechanism (Fig. 6a) showed two minima before reaching the final binding configuration, which corresponds to ensemble A. These two minima were representative of DADME in the gate pointing towards the solvent and towards the binding site, respectively (see also Fig. 5 for a structural representation of these intermediate states). The final state corresponded to the crystallographic pose, and the free energy difference between the initial (DADME in the solvent) and the final (the crystal pose) states was estimated to be about 13 kcal mol^−1^, in good agreement with the experimental *K*_i_ of 9 pM. Notably, the barrier for breaking the pivotal H-bond network established between DADME and the residues of PNP catalytic site turned out to be about 10 kcal mol^−1^, in remarkably good agreement with the experimental value reported by Kicska *et al*.^18^ on removal of the bi-dentate interaction.

Figure 6b shows the free energy along the frontal path from the solvent into the catalytic pocket reaching the crystallographic pose (that is, ensemble A). The free energy difference between the bound and unbound states was also in this case about 14 kcal mol^−1^. The similarity of the binding free energy differences obtained along the two different paths, in agreement with what has to be expected from a state function, substantiated the validity of our approach. In the case of the frontal path, the free energy profile showed less intermediate minima compared with the free energy profile associated with the gating mechanism (compare Fig. 6a,b). This was likely due to the lack of complex conformational rearrangements similar to those observed in the gating mechanism of binding.

In Fig. 6c, we report the free energy profile associated with the upper path. This path led preferentially to ensemble C, and as such, we could not observe any deep minima. This result was in a good agreement with the weak interactions DADME established with PNP in ensemble C (see Fig. 3) and with the fast unbinding mechanism we identified running scaled MD simulations (see the next section for details).

### Binding kinetics

**Figure 7:**
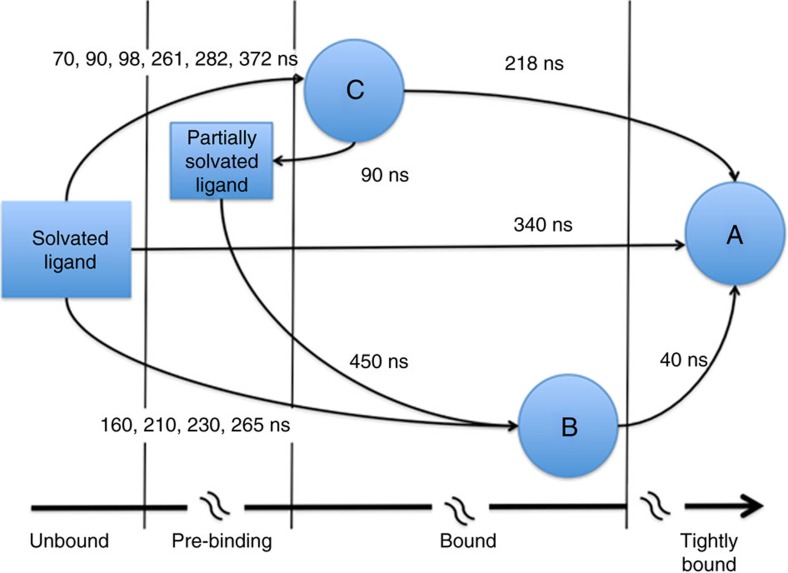
Schematic representation of the association process observed in our simulations. The main different observed states and paths are summarized here, as well as the interconversion times between them. As one can see, the direct binding to the final, long-lived state A was observed only once and took 340 ns. For simplicity, the intermediate state occupied during the ingress into the binding site is indicated only in the case when rebinding was observed.

Analyzing all the binding trajectories, the complexity of the mechanism of DADME binding to PNP clearly emerged (see Fig. 7). Once in 14 different simulations, the ligand directly reached the state A, which corresponds to the crystallographic structure. More often, DADME got stuck in metastable but still inhibitory states B and C, from which it could proceed towards A. However, the ligand often remained in B and C for the entire simulation or left these states for more solvent-exposed configurations and then re-entered, assuming a different metastable configuration, and eventually proceeding to A. In one simulation, DADME took the following route: OUT (full solvation) ->C->OUT (partial solvation) ->B->A (Fig. 7). Although the ligand did not completely escape out of the enzyme, this could be considered a rebinding event^23^. The association process prompted us to provide an estimation of the *k*_on_, as the time for first binding, as reported in the Methods section and discussed below.

The mean first binding time, defined as the expected time, on average, to observe a binding event starting from a situation where all the protein sites are empty, derived from experimental data was estimated to be 246 ns (see Methods for further details). We resorted to the decision of estimating this quantity since it is within the reach of present computational resources and allows us to get a certain statistics on the number of observed events. The expected time for first binding could be an approximation because the long-range interactions between ligand and protein can go beyond the size of our simulation box. We verified this by calculating the electrostatic potential around the trimer and in the proximity of the binding site, as computed by solving the Poisson–Boltzmann equation (see Supplementary Discussion and Supplementary Fig. 1 for details). A strong electrostatic field drives the ligand into the binding site. The isosurface at −1 *k*_B_*T*/*q* intersects the MD simulation box, and therefore an attractive (the ligand carries a positive charge) long-range electrostatic effect can also be present outside of the box, which could increase the local concentration of ligand and therefore reduce the actual time required for binding. Despite these approximations, the simulated mean first binding time, namely the time needed to go from the solvated state to either ensemble A, B or C, was estimated to be 216±101 ns, in fairly good agreement with the value obtained from the available experimental data (that is, 246 ns).

The residence times that characterize potent binders are beyond the reach of present MD simulations, and, as expected, we were not able to observe any spontaneous full unbinding event in our plain MD runs. To identify preferential dissociation routes and to provide a relative estimate of rate constants (*k*_off_ values), we ran biased simulations. In detail, we used scaled MD^16^ and launched six different runs starting from representative structures of A, B and C (see Methods for the simulation set-up). The trajectories starting from A ended up in the fully solvated state in >130 ns of scaled MD. Those from B ended up in the A state in about 20 ns. Finally, starting from C, the unbound state was reached in about 40 ns. We emphasize that in this kind of simulations, exit time can only be considered as a relative quantity, being its sensitivity to the energetic scaling approximately of exponential nature. However, this clearly showed that state A was more stable than state B, which in turn was far more stable than C. B and C can be considered intermediate or alternative states, in which DADME could be trapped during its route towards the final binding configuration. In contrast, in state A, the ligand could establish the tight binding observed in the crystal of the DADME–PNP complex.

### Mechanistic hypothesis

A two-step binding process is used to provide a mechanistic explanation of the experimentally observed slow-onset inhibition^14^. According to this hypothesis, a loose encounter complex is first formed followed by some rearrangements of the system, which strengthen the binding. Our simulations insert this mechanism into a more complex scenario, where three different co-existing binding phenomena interplay. Here the most likely binding leads to ensemble C, where a loose binding complex is observed, and none of the main interactions characterizing the tight binding can be identified. In our simulations, this mesostate was temporarily occupied and most likely, but not necessarily, was followed by an unbinding event. The second most probable occurrence is the binding in the B state, which is more stable than the C state and from which the ligand could evolve to the A state (after 40 ns in one of our plain MD simulations). Direct binding to ensemble A (that is, leading to a crystallographic-like structure without spending much time in intermediate configurations) was statistically much less likely. In addition, we also observed a rebinding phenomenon, where DADME left the C state towards the solvent, and then rebound PNP into the B configuration. Rebinding is a frequent occurrence in protein–ligand binding^23^, but never observed systematically in MD simulations. Based on the present simulations, we hypothesize that the slow-onset inhibition mechanism could be due to a combination of frequent binding to the mesostate C, leading to unbinding and possibly also to rebinding and less frequent binding to B, which could eventually lead to a conformation in the ensemble A. Conversely, direct binding to A is highly improbable and represents the only state in which DADME was tightly bound to PNP, in agreement with the crystallographic structure. In conclusion, a complex interplay of different (re)binding routes and intermediate states could be at the basis of the slow-onset inhibition mechanism experimentally observed for DADME binding to PNP^14^.

Studying protein–ligand binding mechanisms from a thermodynamic and kinetic standpoint can be of paramount importance in drug discovery. MD simulation is emerging as an effective tool for dynamically investigating this process. In this context, we elucidated the full mechanism of binding of DADME, a TSA inhibitor, to the PNP enzyme. The simulations used both unbiased and biased MD approaches. Unbiased MD simulations were used to study the spontaneous binding of DADME to PNP. The mechanism of DADME–PNP binding could be very complex, with several mesostates and pre-binding states where the ligand could be trapped, preventing it from reaching the catalytic pocket where DADME establishes tight interactions with the enzyme. The binding trajectories were analyzed with an *ad hoc* protocol, which includes a customized, unsupervised clustering approach. As a result, three independent binding routes were identified, leading to different PNP–DADME binding configurations. These simulations and the statistics they generated provided an estimation of the *k*_on_, which was in fairly good agreement with the available experimental data. As for the *k*_off_, we could not observe any spontaneous unbinding event, in agreement with the experimental residence time of DADME within PNP of about 20 min. We, however, biased the simulations by carrying out scaled MD runs to observe the relative residence time of DADME in the three ensembles (A, B and C) generated by our clustering approach, and corresponding to the most populated binding configurations. Unbinding from A required more time than unbinding from C, while all the escape routes from B led to A, in both unbiased and scaled MD simulations. Based on this complex and multistep scenario, we could advance a hypothesis for the slow-onset inhibition mechanism shown by DADME with PNP. In particular, the low probability of a direct binding to A, along with the interplay of metastable and pre-binding states, could provide an atomistic explanation of the phenomenological inhibition mechanism experimentally observed for DADME. Interestingly, in one complex binding route, DADME first bound to the C state, then underwent a partial unbinding, then bound back to the protein in B, and finally moved to the tightly bound A state.

It is not surprising that the dynamics of ligand–protein binding can be characterized by a fairly intricate interplay of events, encompassing diverse binding routes and more than just a single binding configuration. Possibly, future experimental techniques will be able to provide more detailed dynamical representations of the binding process. In the meanwhile, and despite the limitations of the current computational approaches, we believe that simulation can be a useful complementary tool to be used in synergy with experiments. As an example, we show that the concerted use of plain and biased MD methods and machine learning can help capture relevant aspects of the mechanisms of biomolecular recognition and interaction.

## Methods

### MD simulations

The methods used for simulations include plain and biased MD, *ab initio* quanto-mechanical calculations for ligand and phosphate charge parameterization and Poisson–Boltzmann equation for electrostatic characterization. For minimization and equilibration, we used the NAMD^24^ code. For production, we used ACEMD^25^. NAMD together with the PLUMED^26^ module were used for running trial simulations involving metadynamics. Gaussian09 (ref. 27) was used for quantum chemical calculations. The PNP homotrimeric unit was simulated in conditions of full phosphate saturation.

The system set-up made use of the AmberTools, version 10 (ref. 28), together with Gaussian^27^ for the ligand and phosphate partial charge computations. The structure of DADME was taken from the crystal corresponding to the PDB entry 1RSZ. Gaussian was used with the HF/6–31G* level of theory, imposing a +1 net charge. The remaining ligand parameters were obtained from the Amber Generalized Force Field (GAFF)^29^ while the RESP procedure (via Antechamber) was used to fit the point charges. The charges on the phosphate group 
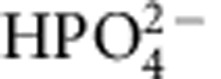
 were obtained with the same settings. The angle parameters were borrowed from a phosphothreonine moiety with a mono-protonated phosphate group present in Professor Bryce’s database of Amber parameters and derived in ref. 30. Bond angle spring for both O2-P-OH and HO-OH-P had a constant of 140 kcal mol^−1^ rad^−2^. This is because the original GAFF parameters made the phosphate hydrogen collapse on the oxygen during the equilibration phase, leading to unphysical forces and failures of the SHAKE algorithm. The equilibrium bond angles were 102.84 and 99.11 degrees for O2-P-OH and HO-OH-P, respectively. The trimer was fully saturated with phosphate groups, which were initially placed in the position induced by the crystal 1RR6 because the crystal of the trimer (PDB entry 3K80) presented a sulfate instead of the phosphate group. A very similar approach was employed for Acyclovir parameterization, with a null net charge and by addition of a suitable number of counterions to neutralize the simulation cell.

The human PNP trimer was obtained from the PDB entry 3K80, and all the simulations were carried out using the trimeric form of the enzyme. PDB entry 3K80 contains two hPNP trimeric subunits. Therefore only chains E, Q and Y were retained. It was first preprocessed by MolProbity^31^ and then His257 was imposed to be of type epsilon as suggested in ref. 10. The first residue (a glutamic acid) was missing from both chains E and Q. It was added back by superimposition with respect to the Y chain. Including the three phosphate groups, the overall charge of the system was of −9 electrostatic unit of charge. To increase chances of observing a binding event, we added to the system nine fully solvated DADME ligands (+1 net charge each), which also did not require the presence of counterions for neutralization of the box. For all the simulations, the Amber99SB-ILDN force field parameters^32^ were used for the protein, while GAFF was used for the ligands. The TIP3P water model was always used.

The system was equilibrated in five phases: the first was done in the NVT ensemble (i.e. constant number of particles, volume and temperature) for 150 ps, with all heavy atoms constrained, except those belonging to waters and ligands, using a harmonic constant of 40 kcal mol^−1^ Å^−2^. A second NVT phase of 50 ps with constrained backbone (harmonic constant of 20 kcal mol^−1^ Å^−2^) was followed by a third NVT phase of 50 ps again with constrained backbone but with a harmonic constraint of 10 kcal mol^−1^ Å^2^. The fourth NVT phase lasted 50 ps with constrained backbone and a harmonic constraint of 1 kcal mol^−1^ Å^−2^. The last phase consisted of 350 ps in the NPT ensemble (i.e. constant number of particles, volume and temperature) and a target pressure of 1 bar.

For the production run, we used ACEMD^25^ with 2 fs time step, switching distance of 7.5 Å, cutoff of 9 Å, electrostatic contribution evaluated every 2 steps, Particle Mesh Ewald with 1 Å spacing, NVT ensemble and Langevin thermostat with 300 K as target temperature and damping of 0.1 ps^−1^. The final box had a size of 11,086,108 Å^3^ and contained nearly 100,000 atoms.

For each of the 14 replicas, the ligand positions were randomly initialized outside the protein (analogously for the five acyclovir replicas). Once the first replica was equilibrated, the others were obtained by performing a steered MD with NAMD and PLUMED of the equilibrated system, aiming to target initial positions of the ligands.

### Machine learning

The machine learning contribution to this work is twofold, consisting in the clustering protocol and in the non-linear interpolation for free energy estimation.

For clustering, we combined a k-medoids algorithm with an effective cluster initialization^12^. This technique has several advantages: it directly minimizes a functional which has a clear geometric meaning, it delivers directly the centroids as samples (frames) belonging to the dataset (the trajectory) and, finally, it employs a robust initialization procedure that pushes the algorithm towards good local minima (good partitioning of the samples). The RMSD of the heavy atoms belonging either to the binding site or to the ligand was used as clustering metric. RMSD was calculated for the possible frame pairs to avoid privileging any particular frame configuration. The effectiveness of the initialization method was supported by the reproducibility of the clustering results, which were independent of the frame order. The final number of clusters was automatically selected by taking inspiration from the elbow criterion on the cost function^33^: the clustering algorithm is run with an increasing number of clusters and for each clustering the total cost (RMSD based) is computed. When the relative decrement of the cost falls <1%, the process stops and the last clustering is kept as final result. As a technical note, the code was optimized and parallelized via multithreading.

For the free energy estimation task, we used Regularized Least Squares^13^ algorithm employing a Gaussian kernel to nonlinearly interpolate the mean force and obtain a smooth free energy. The sigma of the Gaussians was set to 25 and a Tikhonov regularization^34^ coefficient of 1e−8 was used to ensure numerical stability of the solution of the associated linear system of equations. These settings allowed to get a smooth, but still fitting to data, free energy curve.

### First binding time estimation

To get an estimate of the average first binding time, we first derived the *k*_on_ from the experimentally known *k*_off_ and *K*_i_. Then, we used a simple first order reaction model, and its associated differential equation, to extrapolate the short time behaviour of the reactant’s concentration.

Let P, L and C be the molar concentrations of protein, ligand and complex in time. Simple first order reaction equation states:




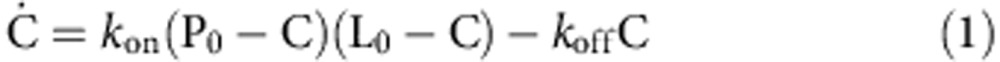




where dotting stands for time differentiating and 0 subscript indicates initial concentration. If one considers the initial derivative and its finite difference approximation, one gets:




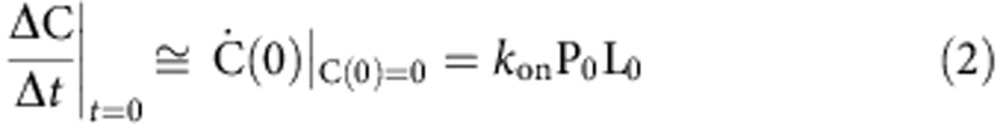




The concentration variation corresponding to one binding event is 1/(*N*_A_*V*_cell_), where *V*_cell_ is the volume of the simulation cell and *N*_A_ is the Avogadro’s number. Considering that in our case, we have three binding sites per system, P_0_ reads 3/(*N*_A_*V*_cell_). Similarly, L_0_ is given by 9/(*N*_A_*V*_cell_). Plugging all these values and inverting (Equation 2) with respect to Δ*t*, one gets:




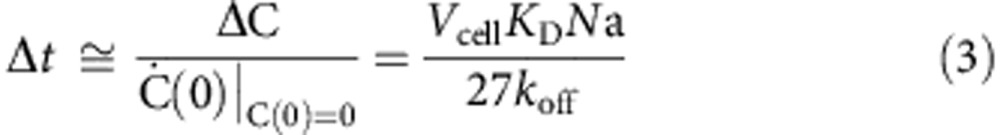




We included in our system nine ligands to reduce the first binding time and neutralize the cell. The cell size was set to 11,086,108 Å^3^ and the thermodynamic and kinetic constants for DADME were *K*_D_=9 pM and 
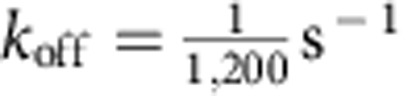
 (inverse half-life). The application of Equation 3 leads to our derived average first binding time of 246 ns. We therefore ran simulations for a minimum of 500 ns each, so as to have a reasonable probability of seeing a binding event during simulation.

### Free energy estimation

The overall methodology consists of four algorithmic steps. In the first, the umbrella centres on the path S-variable are set so that they are as equidistant as possible. Then, the mean force on each point is estimated via direct integration of the force over time and the overall mean force profile as a function of the S-variable is reconstructed using non-linear least squares. Finally, the free energy is computed as minus the integral of the mean force, that is, from the definition.

Sample configurations of the binding process were taken from the three trajectories that best represented the three main binding paths. The umbrella centres were manually selected among these configurations. We would like to highlight that the sample resolution required to reconstruct the free energy profile along a reactive path is higher than that needed to perform a clustering procedure. This is because the latter provides information on the free energy basins (that is, high probability regions), which are more largely populated. The former rather also needs sampling on much less populated regions, such as those neighbouring the TS. This was the reason to do a manual selection of the centres. A further reason was represented by the technical requirement of having equidistant centres due to the S-path collective variable definition.

To check the hypothesis that the spontaneous binding routes observed in our plain MD were close to actual minimum free energy paths, we also sampled the Z-variable (which is orthogonal to the S-variable used to move along the path) during the umbrella sampling. We verified that the *Z* values were mostly very small (<0.01 Å^2^), confirming our initial expectation.

The spring constants for the harmonic restraints for the umbrella sampling were set to 500 kJ mol^−1^. We used 11, 17 and 27 approximately equidistant umbrella centres for the upper, frontal and gate paths, respectively. Consistently with the distance between the umbrella centres, the lambda values were set to 100 nm^−2^ for the gate and frontal path and 50 nm^−2^ for the upper path, the underlying metric was MSD and the selected atoms were the same as those used for clustering. Plumed2 (ref. 35) was used to impose the constraints at the umbrella centres. Mean force was estimated using the usual expectation procedure (see Maragliano *et al*.^22^). Consistency of mean force estimation was checked by the in-sample bootstrapping method.

## Additional information

**How to cite this article**: Decherchi, S. *et al*. The ligand binding mechanism to purine nucleoside phosphorylase elucidated via molecular dynamics and machine learning. *Nat. Commun.* 6:6155 doi: 10.1038/ncomms7155 (2015).

## Supplementary Material

Supplementary Figures, Discussion, Methods and References Supplementary Figures 1-8, Supplementary Discussion, Supplementary Methods and Supplementary References.

Supplementary Movie 1 Dihydroxypyrrolidine ring of DADME in the binding site of PNP loosing the flipped pose and reaching the crystal-like configuration.

Supplementary Movie 2 DADME entering into the PNP binding site through the "upper" path.

Supplementary Movie 3 DADME entering into the PNP binding site through the "frontal" path.

Supplementary Movie 4 DADME entering into the PNP binding site via the "gating" mechanism and reaching the final crystal-like configuration after spending some time in a pre-binding state.
