# Supplementary material for: The ligand binding mechanism to purine nucleoside phosphorylase elucidated via molecular dynamics and machine learning
Source: Nat Commun. 2015 Jan 27;6:6155. doi: 10.1038/ncomms7155 (PMC4308819; doi:10.1038/ncomms7155)
Supplement: Supplementary Figures, Discussion, Methods and References — Supplementary Figures 1-8, Supplementary Discussion, Supplementary Methods and Supplementary References. [file ncomms7155-s1.pdf]

Supplementary Figures

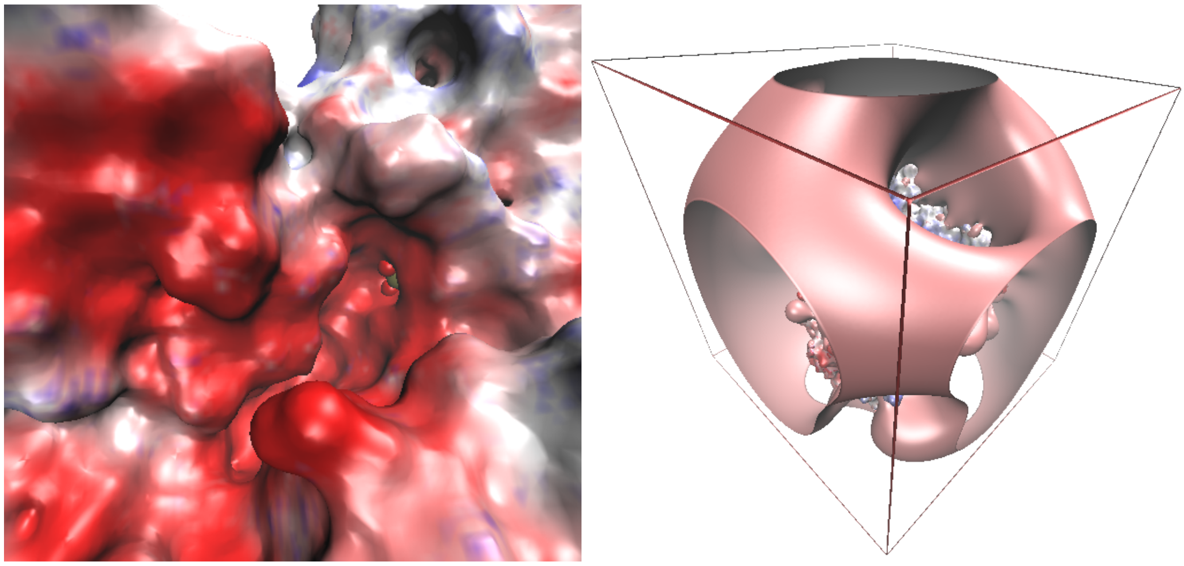

Supplementary Figure 1. The left panel presents PNP surface (binding site detail) computed as a Skin surface colored by the electrostatic potential in the range [-2.0,2.0] kt/q. In the right panel, the isosurface at -1 kT/q is shown together with the simulation box.

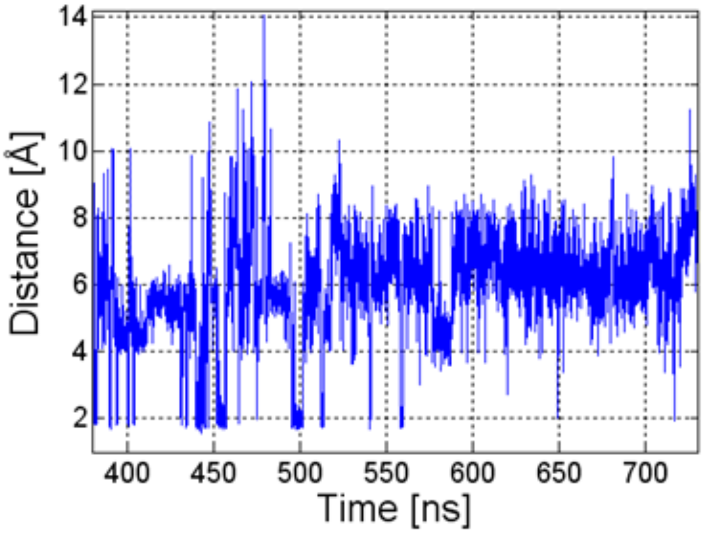

Supplementary Figure 2. Distance between Nδ of His257 and OH of DADME ligand for the best replica.

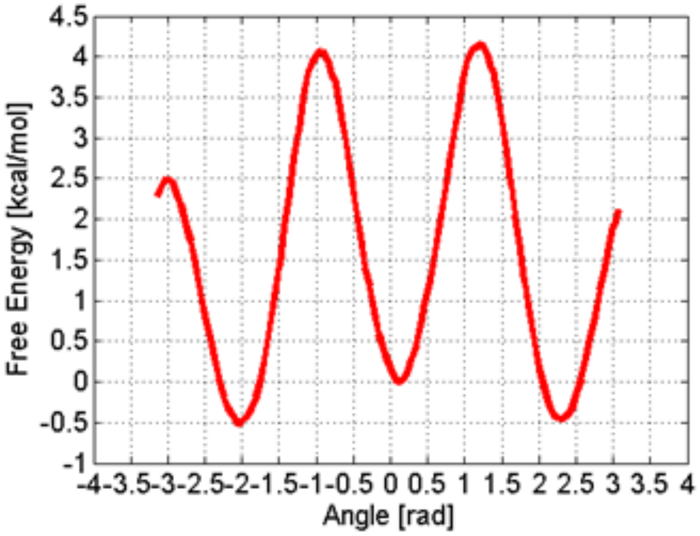

Supplementary Figure 3. Free energy in water of DADME with respect to the upper dihydroxypyrrolidine-purine dihedral angle. The crystal minimum is at 0 rad for clarity of representation.

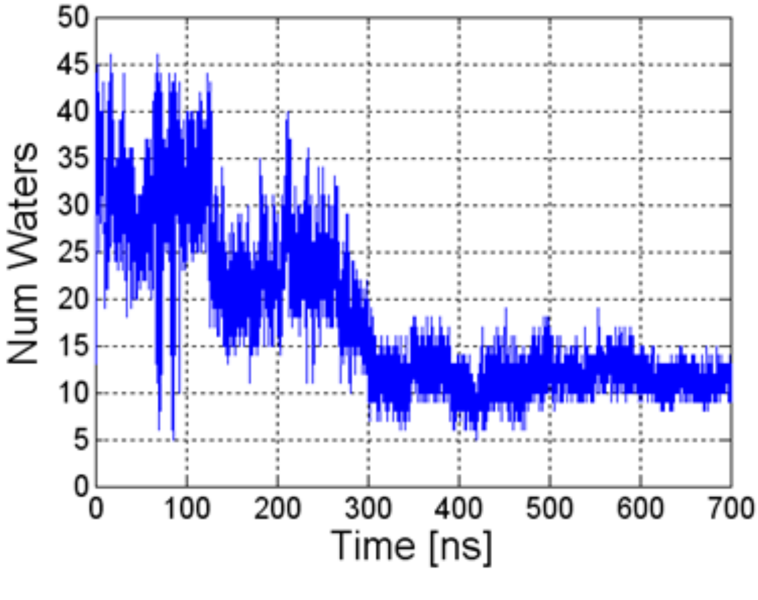

Supplementary Figure 4. Solvation, in terms of number of water molecules within 3.5 Angstrom of the ligand, from the bulk to the binding event.

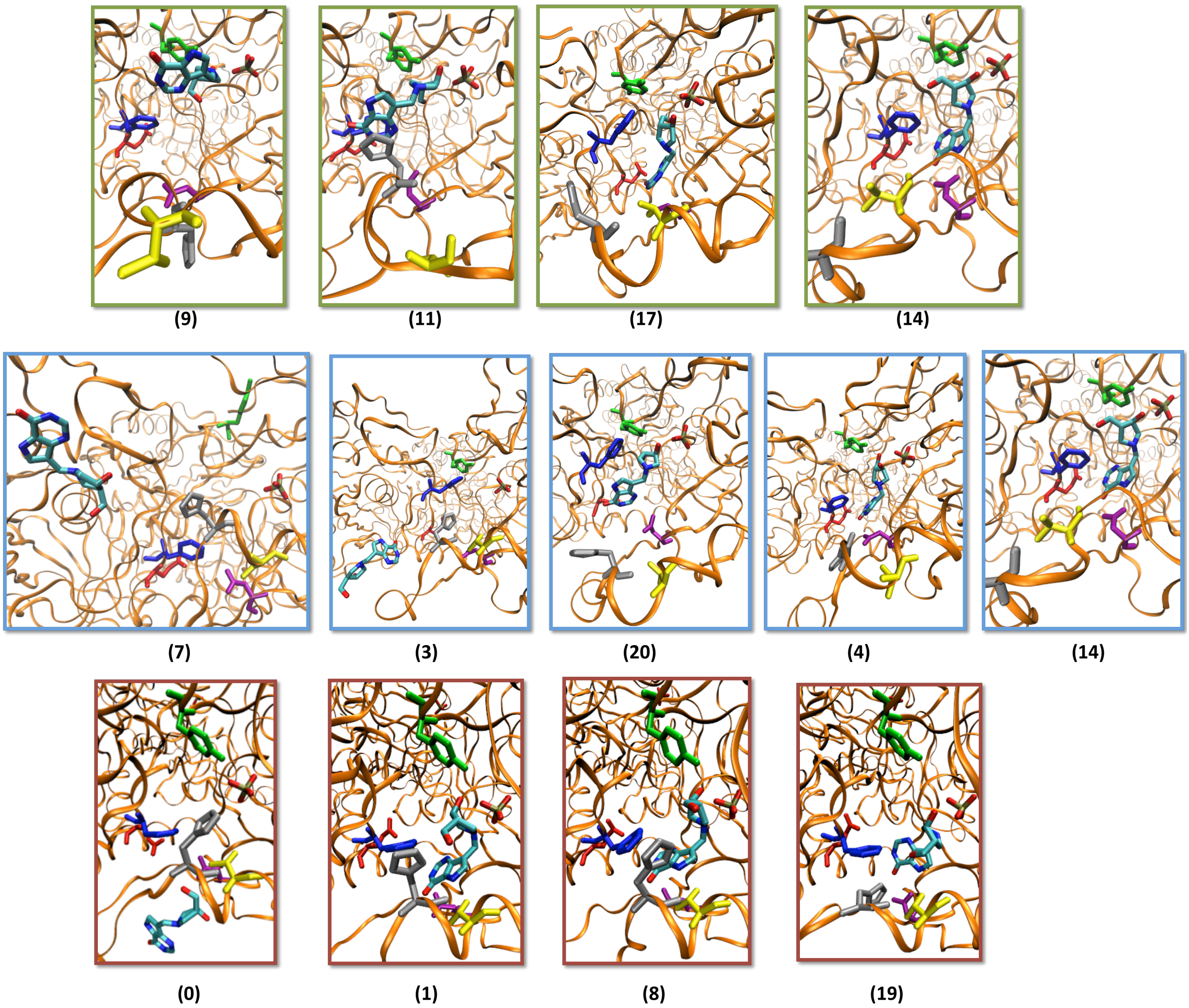

Supplementary Figure 5. In each row, a binding path is represented. Colors of the frames encode the corresponding path. The cluster index is between braces.

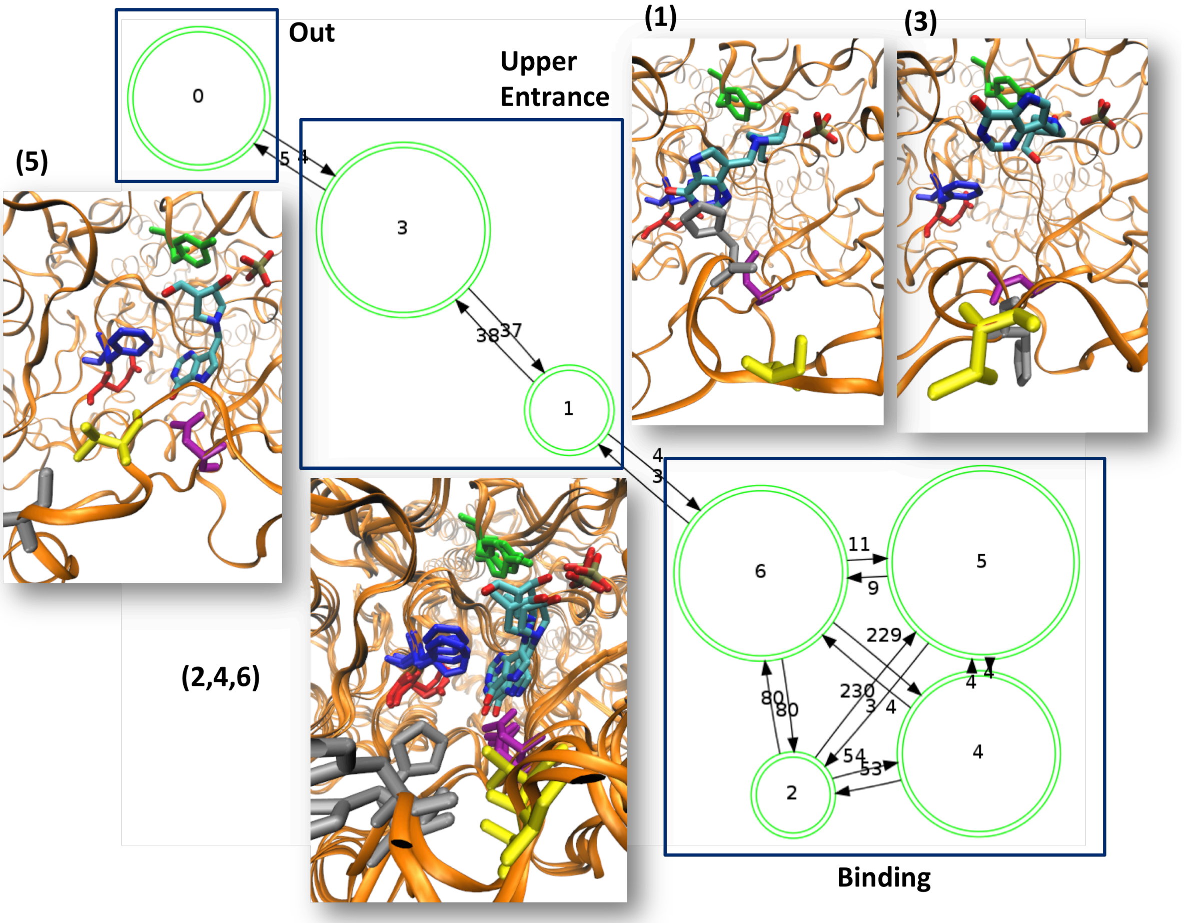

Supplementary Figure 6. Clustering for the upper entrance path. Clusters results are shown together with the medoids poses. Circle sizes are proportional to the cluster sizes. On each edge, the number of transitions between connected clusters without imposing the detailed balance is reported. In green Tyr88, in magenta Asn243, in yellow Val260, in blue Phe200, in red Glu201, in silver His257, and in standard atomic color coding, the ligand and the phosphate.

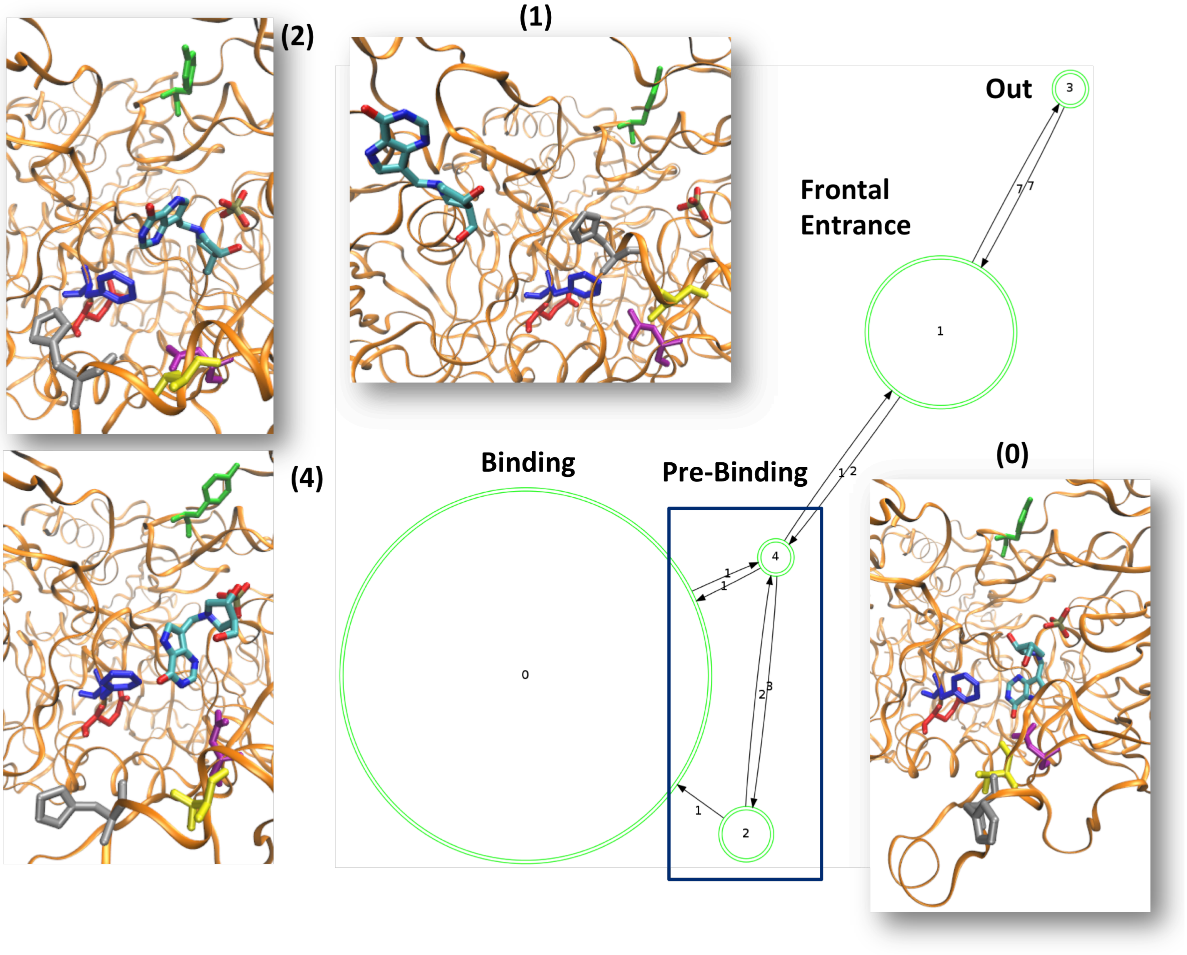

Supplementary Figure 7. Clustering for the frontal entrance path. Clusters are shown together with the medoids poses. Circle sizes are proportional to cluster sizes. On each edge, the number of transitions between connected clusters without imposing the detailed balance is reported. In green Tyr88, in magenta Asn243, in yellow Val260, in blue Phe200, in red Glu201, in silver His257, and in standard atomic color coding, the ligand and the phosphate.

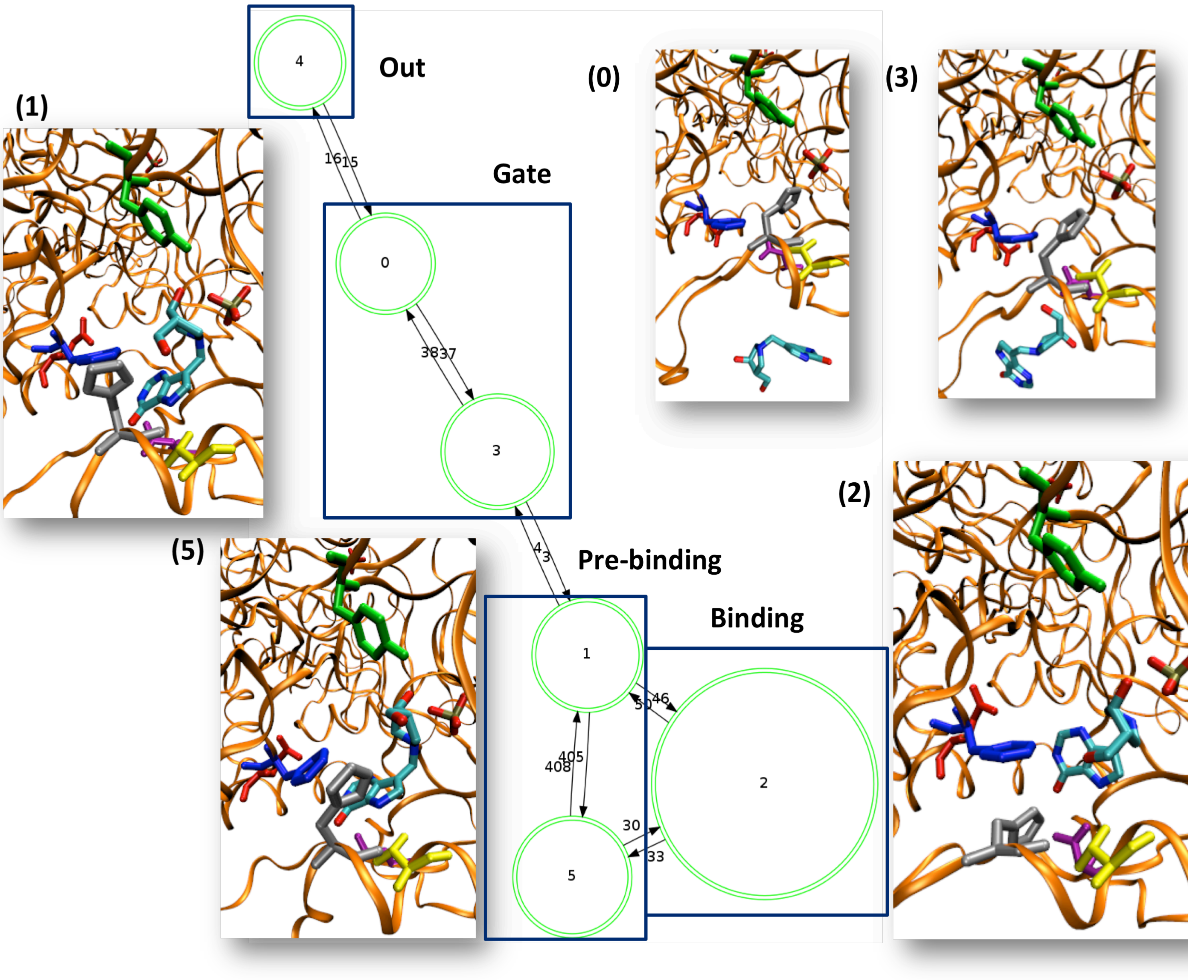

Supplementary Figure 8. Clustering for the gate path. Clusters results are shown together with the medoids poses. Circle sizes are proportional to the cluster sizes. On each edge, the number of transitions between connected clusters without imposing the detailed balance is reported. In green Tyr88, in magenta Asn243, in yellow Val260, in blue Phe200, in red Glu201, in silver His257, and in standard atomic color coding, the ligand and the phosphate.

Supplementary Discussion

Electrostatic environment around the PNP trimer. To characterize the electrostatic field generated by PNP, we used a DelPhi Poisson-Boltzmann equation solver[2]. We used a grid spacing of 2 $Å^{-1}$, a volume occupation of 80%, Coulombic boundary conditions. The interior dielectric constant was set to 2 and the external one was set to 80. We used the same radii and charges as in the MD simulation. The molecular surface used for calculations was the Connolly implementation of the NanoShaper module[3]. For visualization purposes, the Skin surface was computed by NanoShaper and imported into VMD. NanoShaper and related scripts to carry out the conversions and computations are available at www.electrostaticszone.eu.

As made clear by the isosurface of the potential at -1kT/q (Supplementary Fig. 1), the effects of the charge on the trimer go beyond the size of the simulation cell.

Role of His257 in DADMe-Immucillin-H binding. Schramm and coworkers[4] discussed the role of His257 in binding based on the results of several experiments. Mutating His257[4] does not hinder binding of DADMe-Immucillin-H (DADME). It only slightly reduces affinity.

In our binding simulations, we monitored the existence of an H-bond between the -OH of ribose of DADME and the delta Nitrogen of His257. We found that our simulated binding events exhibit the same stability, evaluated via RMSD, irrespective of the interaction with His257. In particular, we closely inspected this H-bond in the trajectory that led to the closest conformation to the crystal-binding pose (Supplementary Fig. 2). During that binding trajectory, the H-bond continuously broke and reformed, which is consistent with previously reported experimental results and simulation findings[5].

The same behavior was observed when simulating the complex, starting from the crystal configuration. Interestingly, and as already observed by Hirschi et al.[5], the interaction with His257 is quite rapidly lost in 10 ns of simulation. In longer simulations, this interaction takes place with an intermitting pattern.

His257 might play a role more related to the early stages of binding. In particular, in one of our binding trajectories, Asn243 stabilizes the purine by double H-bonding with N7-H and O6. Interestingly, during the pre-binding phase, the N7-H H-bond is replaced by another H-bond with the oxygen of His257. This makes us conjecture that, in the early binding stage, His257 plays a role that is similar to but weaker than that played by Asn243 in purine stabilization. Later, when the ligand completely enters the site, Asn243 keeps the purine rings locked.

Exploring conformational minima in solution. We performed a well-tempered metadynamics run on dihydroxypyrrolidine-purine dihedral by employing the Plumed plug-in with NAMD. For both, Gaussians height was set to 0.05 kcal/mol, simtemp was set to 300 K, the bias was set to 12, deposition occurred every 1000 integration steps (every 2 ps) and sigma was set to 0.05 rad. In Supplementary Fig. 3, the minimum at 0 radians represents the crystal pose, while the left minimum represents the flipped one. In solution, the flipped pose is

more probable than the crystal one: this explains why DADME is more likely to enter into the binding site in the flipped than in the crystal conformation (ensemble B of the main text).

Dehydration. Supplementary Fig. 4 reports the temporal evolution of solvation in terms of number of waters within 3.5 Å of the ligand; these data refer to the simulation that led to the best binding pose with respect to the crystal. The obtained values are comparable but slightly larger (7 vs. 10) than those shown in ref.5. The transient part of the graph represents the phase in which the ligand gets anchored to the alpha-helix/loop located at residues 240-260 where the number of water molecules is around 20. Upon binding, the number of water molecules quite rapidly drops down to about 10. Correspondingly, upon 'gate opening', the RMSD of the binding site residues slightly changes to accommodate the ligand.

Clustering. Supplementary Fig. 5 reports the snapshots corresponding to each cluster (the numbering is related to the main text) along the binding paths. Supplementary Figures 6, 7, and 8 report the clustering results on the path subsets of frames together with the image of the centroids.

Negative-control simulations. To test the robustness of our results, we simulated five further replicas of the PNP enzyme for a total simulated time of 2.5 µs, 500 ns each. In these runs, PNP was simulated with the drug acyclovir[6], a molecule with known micromolar affinity for the PNP enzyme. The parameterization of acyclovir was carried out in the quantum mechanical framework at the 6-31G* level of theory (the same protocol used for DADME) followed by RESP fitting to estimate partial charges. The MD simulation protocol was identical to that previously used for DADME. These simulations led to the following observations. i) In one out of five simulations, acyclovir managed to get into the PNP binding pocket; the observed bound configuration had an RMSD of 2.8 Å compared to the crystal structure 1PWY. This confirms that the procedure allows a reasonable pose to be achieved in the allowed time of 500 ns. ii) The frontal path was followed by acyclovir during that binding. This is consistent with and similar to one of the paths observed for DADME when binding to PNP. This supports the reasonable hypothesis that binding paths can be common to different ligands. iii) Furthermore, the occurrence of only a single binding event confirmed this ligand had lower affinity than DADME (micromolar vs. subnanomolar). We should comment here that available experimental data were only on affinity and not on kinetics rates. It can however be expected that the difference in affinity is due to both a slower k_{on} and a faster k_{off}. In this framework, these results strengthen the reliability of the main outcomes of the paper, concerning DADME binding to PNP.

Supplementary Methods

Initialization of ligand positions via steered MD simulations. Steered MD simulations were performed via Namd 2.8 together with Plumed 1.3. We built 14 replicas of randomly positioned ligands at least 5 Å from the protein. We used these positions as independent starting configurations. Each was used as a target for the steered MD. In Plumed, the collective variable MSD was used with NO_ROT and NO_CENTER specifications to assure movement to the absolute positions. We used a spring constant value of 50 kcal/mol/$Å^2$ and a velocity of 0.5 Å/ps.

Stability check of the crystal complex. To support the validity of the parameters adopted in our simulation, the stability of the crystal structure of the complex was assessed. We simulated the monomer structure 1RSZ with DADME for 200 ns with Acemd[1] and checked the stability of the binding conformation. The RMSD of the ligand heavy atoms during the simulation remained within the range of 1.1-1.52 Å of RMSD with respect to the original crystallographic conformation.

Stability of the APO form of the PNP trimer. The main aim of this simulation was to estimate the flexibility of the alpha helices of the PNP trimer in the absence of the ligands. The trimer (pdb code 3K8O) was simulated for 100 ns with no constraints, employing Acemd, after removal of the ligands. We observed that, at the end of the simulation, two of the three alpha helices lost the kink. We also observed that formation and loss of the kink is reversible and that the alpha helix is characterized by high mobility. This is confirmed by the high beta factor of that region in the crystal structure deposited in the Protein Data Bank.

Supplementary References

1 Harvey, M. J., Giupponi, G. & Fabritiis, G. D. ACEMD: Accelerating Biomolecular Dynamics in the Microsecond Time Scale. *Journal of Chemical Theory and Computation* **5**, 1632-1639, (2009).

2 W. Rocchia, E. A., B. Honig. Extending the Applicability of the Nonlinear Poisson-Boltzmann Equation: Multiple Dielectric Constants and Multivalent Ions. *J. Phys. Chem. B* **105**, 6507-6514, (2001).

3 Decherchi, S. & Rocchia, W. A general and robust ray-casting-based algorithm for triangulating surfaces at the nanoscale. *PLoS One* **8**, e59744, (2013).

4 Murkin, A. S. *et al.* Neighboring group participation in the transition state of human purine nucleoside phosphorylase. *Biochemistry* **46**, 5038-5049, (2007).

5 Hirschi, J. S., Arora, K., Brooks, C. L., 3rd & Schramm, V. L. Conformational dynamics in human purine nucleoside phosphorylase with reactants and transition-state analogues. *J Phys Chem B* **114**, 16263-16272, (2010).

6 Marangoni dos Santos, D. *et al.* Crystal structure of human purine nucleoside phosphorylase complexed with acyclovir. *Biochemical and Biophysical Research Communications* **308**, 553 - 559, (2003).
